# Association of Reactive Oxygen Species-Mediated Signal Transduction with In Vitro Apoptosis Sensitivity in Chronic Lymphocytic Leukemia B Cells

**DOI:** 10.1371/journal.pone.0024592

**Published:** 2011-10-10

**Authors:** Adam L. Palazzo, Erik Evensen, Ying-Wen Huang, Alessandra Cesano, Garry P. Nolan, Wendy J. Fantl

**Affiliations:** 1 Nodality Inc., South San Francisco, California, United States of America; 2 Baxter Laboratory for Stem Cell Biology and Department of Microbiology and Immunology, Stanford University, Palo Alto, California, United States of America; The University of Birmingham, United Kingdom

## Abstract

**Background:**

Chronic lymphocytic leukemia (CLL) is a B cell malignancy with a variable clinical course and unpredictable response to therapeutic agents. Single cell network profiling (SCNP) utilizing flow cytometry measures alterations in signaling biology in the context of molecular changes occurring in malignancies. In this study SCNP was used to identify proteomic profiles associated with *in vitro* apoptotic responsiveness of CLL B cells to fludarabine, as a basis for ultimately linking these with clinical outcome.

**Methodology/Principal Finding:**

SCNP was used to quantify modulated-signaling of B cell receptor (BCR) network proteins and *in vitro* F-ara-A mediated apoptosis in 23 CLL samples. Of the modulators studied the reactive oxygen species, hydrogen peroxide (H_2_O_2_), a known intracellular second messenger and a general tyrosine phosphatase inhibitor stratified CLL samples into two sub-groups based on the percentage of B cells in a CLL sample with increased phosphorylation of BCR network proteins. Separately, in the same patient samples, *in vitro* exposure to F-ara-A also identified two sub-groups with B cells showing competence or refractoriness to apoptotic induction. Statistical analysis showed that *in vitro* F-ara-A apoptotic proficiency was highly associated with the proficiency of CLL B cells to undergo H_2_O_2_-augmented signaling.

**Conclusions/Significance:**

This linkage in CLL B cells among the mechanisms governing chemotherapy-induced apoptosis increased signaling of BCR network proteins and a likely role of phosphatase activity suggests a means of stratifying patients for their response to F-ara-A based regimens. Future studies will examine the clinical applicability of these findings and also the utility of this approach in relating mechanism to function of therapeutic agents.

## Introduction

CLL is the most common adult leukemia in the Western world and is characterized by aberrant accumulation of CD5+ B lymphocytes in the peripheral blood, bone marrow and secondary lymphoid organs. Clinical presentation, natural course of the disease and response to treatment are all extremely variable, with patient survival after diagnosis ranging from months to decades. Although the biological mechanisms accounting for the unpredictability of the disease are unknown, several biological indicators including cytogenetics, presence or absence of somatic mutations within the immunoglobulin heavy chain variable region (IgV_H_), ZAP70 and CD38 expression have all been associated with response to therapy and prognosis [1]–[3].

A greater understanding of CLL biology is needed to chart disease progression as well as assist in selecting optimal therapeutic strategies. Ideally, monitoring on an individual patient basis would take into account differing inter-patient cell biology as well as shifts in the intra-patient population biology of mutant cells within a heterogeneous tumor cell population. SCNP studies in myeloid leukemias and follicular lymphoma distinguished healthy from diseased cells by their response to growth factors and cytokines [4]–[10]. In these studies induced protein phosphorylation was shown to be more informative than the frequently measured basal phosphorylation state of a protein revealing signaling deregulation consequent to the numerous cytogenetic, epigenetic and molecular changes characteristic of transformed cells. Furthermore, SCNP simultaneously measures multiple signaling proteins and assigns their activation states to specific cell sub-sets within complex primary cell populations [11].

Central to B cell development, and also believed to be important in CLL progression, is the BCR signal complex composed of membrane-bound immunoglobulin and the signal transducing CD79α/CD79β heterodimer. In normal B cells, antigen mediated BCR activation regulates cell survival, differentiation, proliferation and migration [12], [13]. Additional regulation of BCR signaling involves phosphatase(s) whose activity is regulated by NADPH-oxidase-generated reactive oxygen species H_2_O_2_ [14]–[16]. Furthermore, studies from the groups of Munroe and Rajewsky have recognized that in conjunction with antigen-driven responses, ligand-independent signaling (tonic signaling) by both the pre-B cell receptor and BCR has an important role in survival throughout B cell development [17]–[20]. Although the molecular mechanisms governing tonic BCR signaling are not well defined, recent studies suggest that tyrosine phosphatase regulation by reactive oxygen species play a likely role [14], [16], [17], [21]–[23]. Furthermore, recent evidence has described deregulated tonic BCR signaling in diffuse large B cell lymphoma and CLL [24]–[28].

In CLL, associations have been observed between the clinical course of the disease and functional alterations in the BCR and its regulators, suggesting that both antigen-driven and tonic BCR signaling play an important role in its pathogenesis. This is corroborated by *in vitro* studies in which significant differences in both ligand-mediated and ligand-independent BCR signaling were found in primary CLL patient samples [25], [29].

In a healthy physiological setting, apoptosis proceeds from sensors that monitor cell stress and damage, to effectors that relay the signals to activate programmed cell death pathways. Apoptosis is also regulated by cell survival signals, and in B cells one such set of signals emanates from the BCR via tonic signaling, as noted above. The accumulation of malignant monoclonal B cells in CLL has largely been attributed to defects in apoptosis cascades rather than to aberrant proliferation [30]. In some CLL patients inactivation of cell death pathway proteins such as p53 (17p deletion) is an example of how this mechanism can over-ride benefit from a therapeutic agent [31]. No relationship between BCR signaling, cell survival and resistance of patient cells to chemotherapy has yet been shown using existing analytical methods. SCNP technology now provides an opportunity to re-examine the global alterations in signaling that inevitably occur in B cells in response to the genetic and molecular changes they have sustained.

## Materials and Methods

### Ethics statement

All patients consented, in accordance with the Declaration of Helsinki, for the collection and use of their samples for institutional review board-approved research purposes.

### Patient samples

Cryopreserved, Ficoll-purified (Sigma Aldrich) [32] peripheral blood mononuclear cells (PBMCs) from 23 CLL and 7 healthy subjects were used in this study (Table 1). The majority of samples were collected from patients previously treated. CLL diagnosis was based on the Workshop on Chronic Lymphocytic Leukemia criteria [33].

**Table 1:** Clinical and molecular characteristics of patient samples.

Sample ID	Gender	Age at DX	IgVH	% ZAP 70	FISH	First Treatment (Rx) Type
CLL001	**M**	44	93	0.4	Not available	HDMP+Rituxan
CLL003	F	53	92.6	0.6	Not available	FR (2cycles) - good response
CLL004	**M**	53	100	92.5	11qdel(16%)	HDMP Rituxan Frontline (3 cycles)
CLL005	**M**	65	98.6	43.6	tri12 (90%)	Leukeran (4 mg/day)
CLL006	F	61	99.6	45.2	normal FISH	F; then FR (4 cyc) - MRD on BM
CLL007	**M**	66	99.3	79.1	tri 12 (84%)	Leukeran
CLL008	**M**	72	95.8	3	13q del (73%)	Leukeran
CLL009	**M**	81	95.2	9.8	13q del (100%)	Chloroambucil
CLL010	**M**	48	89	5	Not available	Fludarabine (3 cycles)
CLL011	**M**	57	93.3	1.1	tri 12(75%)	ASCENTA-002
CLL012	F	72	96.8	1.2	Not available	no Rx
CLL013	**M**	59	99.6	62.8	tri 12(89%)	Chlorambucil
CLL014	**M**	54	90.5	0.5	tri 12 (karyotype)	High dose chemoRx w/BM transplant
CLL015	F	51	100	91.9	tri 12 (82%)	ASCENTA-002
CLL016	F	53	93	0.1	Normal FISH	HDMP+R 04 Frontline
CLL017	F	39	96.1	10.1	Normal Karyotype	no Rx
CLL018	**M**	55	100	91.1	13qdel(70%)	HDMP+R 04 (3 cycles) Frontline
CLL019	**M**	50	100	45.9	Normal FISH	Rituximab (3 cycles) - 4 weeks
CLL020	**M**	43	91.9	1.1	13 del (99%)	Chlorambucil
CLL021	**M**	66	100	65.3	13 del (99%)	Solumedrol Rituxan
CLL022	**M**	48	100	73.1	tri 12 (80%)	no Rx
CLL023	**M**	72	94.7	1.3	13 del (17%)	GMCSF and Rituxan
CLL024	F	49	100	51.8	Not available	no Rx

IgV_H_ mutational status and percent ZAP70-positive cells determined as previously described [62].

### Cell preparation

Samples were thawed at 37°C and suspended in RPMI 1640 1% FCS. Viability was measured for an aliquot immediately post thaw with trypan blue. In this sample set viability was greater than 90% for all samples. Amine Aqua (Invitrogen) was used to determine cell viability according to the manufacturer’s protocol. Briefly, Amine Aqua was added to all samples before the 2 hour rest and was present throughout the duration of the experimentation. Cells were arrayed in duplicate in 96-deep-well plates at 6.0×10^5^ cells or 8.0×10^5^ cells per well for measurements of BCR signaling and apoptosis respectively. More cells were used for apoptosis assays to get a more reliable measurement over the 48 hours of exposure to F-ara-A. All measurements for signaling and apoptosis were performed in duplicate for each sample and assay performance characteristics noted (manuscript in preparation).

### Ramos cell line control

The human Burkitt lymphoma Ramos cell line, a control for BCR signaling, acquired from ATCC was cultured according to the manufacturer’s protocol.

### Treatment with extracellular modulators of BCR and apoptosis pathways

After a 2 hour rest at 37°C, each sample was treated in bulk for 10 minutes at 37°C with goat polyclonal (F(ab’)_2_ human anti−μ (anti−μ) or anti−γ (Southern Biotech), final concentration 10 µg/ml or with H_2_O_2_, final concentration 3.3 mM. For the combination of anti−μ and H_2_O_2_, anti−μ was added first followed by H_2_O_2_ [16]. Phorbol Myristate Acetate ((PMA) Sigma), final concentration 400 nM, was used as a control to show that cells were capable of signaling, in this case downstream of Protein Kinase C and in a BCR-independent manner. For apoptosis assays, samples were exposed either to 9-β-D-arabinosyl–2-fluoroadenine (F-ara-A, Sigma-Aldrich), the free nucleoside of fludarabine, final concentration 1μM, staurosporine (Sigma-Aldrich), final concentration 5 µM for 48 and 6 hours respectively or to vehicle (0.05% DMSO) for matching times at 37°C [34], [35].

Cells were fixed with paraformaldehyde and permeabilized with 100% ice-cold methanol as previously described [5], [16], [36].

### Flow cytometry determinations of BCR and apoptotic pathways

Cells were incubated with panels of fluorochrome-conjugated antibodies against a core set of B cell phenotypic markers combined with antibodies recognizing intracellular signaling or apoptosis molecules (Table S1). All antibody concentrations were optimized to maximize signal to noise ratio and to minimize non-specific binding. Eleven point titrations (two fold dilutions) were performed with cryopreserved PBMCs from two healthy donors or with the Ramos cell line. For surface antibodies, titrated in PBMCs, the optimal concentration selected is one where the median fluorescence intensity (MFI) of the signal to noise ratio is maximal between a cell immunophenotype that expresses an epitope versus one that does not. For the antibodies against phospho-specific epitopes, antibody titrations were performed to determine the concentration that gave the maximal signal to noise ratio for fold increase of stimulated over unstimulated signal (median fluorescence intensity (MFI) of stimulated/ MFI unstimulated sample) in cell lines and PBMCs (data not shown). Further determinations of phospho-antibody specificity were determined by pre-blocking the antibody with the phospho-peptide epitope against which the antibodies were generated (data not shown).

Samples processed for cytometry [16], [36] were acquired on a BD FACS Canto II flow cytometer equipped with a high throughput sampler (HTS).

### Expression of surface markers on unfixed cells

Cells were incubated with immunophenotypic cocktails (Table S1) before FACS analysis.

### Data analysis

Cells gated on light scatter characteristics were evaluated for viability by exclusion of Amine Aqua. Live cells were gated as CD3-/CD20+ to allow for direct comparison of B cells from CLL and healthy samples. The gating scheme is shown in Figure S1. Metrics included median fluorescence intensity (MFI), percentage of positive cells, and mixture-model derived population content and were extracted from CD3-/CD20+ cells. FCS files were analyzed in FlowJo (Treestar, Ashland, OR) version 8.8.2. To display and compare intensity values including negative numbers and correct for large variance with some fluorophores we used the inverse hyperbolic sine (arcsinh) with a cofactor instead of the traditional log_10_ scale as shown below.

The arcsinh transformation behaves linearly for small values and log-like for larger values. This transformation for fluorescent intensities was computed using the expression:
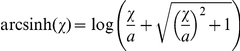



where 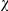 is the fluorescent intensity and 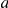 is a factor that determines the linear region of the transform. The addition of 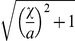 term guarantees that the log function will have a positive value even if 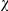 is negative.

The criteria used to assign apoptotic proficiency to a sample were a two-fold or greater increase in the number of CD3^−^−/CD20+ cells at 48 hours that were positive for cleaved PARP (c-PARP+) and cleaved Caspase 3 (C-Caspase 3) after exposure to F-ara-A compared to vehicle or to staurosporine compared to vehicle. The criteria to assign apoptotic refractoriness to F-ara-A were a less than two fold change in the number of of CD3^−^/CD20+ cells at hours that were positive for c-PARP+and c-Caspase3+.

## Results

### Surface marker characterization and basal phosphorylation states in healthy and CLL B cells

**Figure 1:**
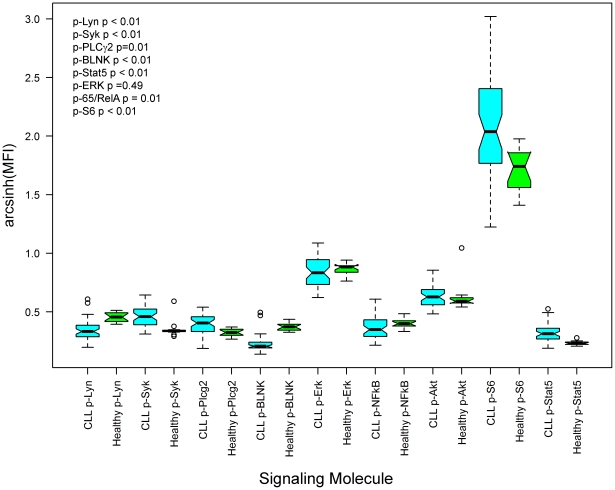
Basal phosphorylation levels of signaling molecules downstream of the BCR. Box and whisker plots comparing magnitude and range of basal signaling between CLL (blue) and healthy donors (green). Notch and red horizontal line indicates median signaling for parameter, box drawn from lower to upper quartiles of data, and whiskers extend to 1.5 times the interquartile range. p-values from Student’s t-test comparing Arcsinh transformed MFI values from CLL and healthy B cells.

Comparison of MFI values of BCR signaling molecules in their basal phosphorylation states showed greater variability in CLL versus healthy B cells. MFI values for p-Akt and p-Lyn spanned a range of 16 and 17 respectively among healthy B cells and 63 and 66 in CLL B cells (Figure 1 and Table 2). p-Erk and p-65/RelA showed no significant differences between healthy and CLL samples, indicating that at their basal level the activation state of these molecules did not reflect a CLL-dependent phenotype. Although not statistically significant, some samples did have high basal levels of p-65/RelA consistent with previous reports documenting a wide degree of variability for the phosphorylation status of this transcription factor [37] [38]. Similarly, expression (determined by MFI) of B cell lineage markers (CD5, CD19 and CD20) and tyrosine phosphatases (CD45, SHP-1 and SHP-2) also showed greater variability in CLL B cells (Table S2). The expected 2∶1 kappa/lambda ratio was evident in healthy B cells and contrasted with the exclusive expression of either kappa or lambda chain in CLL B cells (data not shown). In 4 samples no light chain, IgM or IgG were detected. Although technical limitations cannot be excluded, the presence of CLL B cell markers such as CD5, CD19 and CD20 suggest these cells to be a clonal B cell population with a CLL phenotype. Expected hallmarks of CLL were seen in the low expression of IgM and CD79βin individual patient samples (Table S2). For CD38 expression most samples were negative but in seven samples, there was a bimodal profile for CD38 expression (data not shown and [39]. No significant separation of CLL samples into distinct subgroups could be made based on expression levels of the measured surface markers or the tyrosine phosphatases.

**Table 2:** Range of basal phosphorylation levels (MFI) of BCR signaling molecules in CLL and healthy B cells.

		p-Lyn	p-Syk	p-PLCγ2	p-BLNK	p-Erk	p-65/RelA	p-Akt	p-S6	p-Stat 5
**CLL**	Range	66	62	57	50	94	68	63	1155	43
	Mean	52	79	63	35	153	56	105	579	51
	Stdev	14	16	15	11	28	17	16	287	11
**Healthy**	Range	17	16	17	21	41	19	16	198	6
	Mean	66	55	51	53	157	57	94	377	35
	Stdev	6	5	8	8	13	6	6	72	2

### Modulated signaling responses distinguish subgroups of CLL patient samples

To test whether differences in CLL physiology could be discerned based on intracellular signaling responses, cells were treated with extracellular modulators. The modulators chosen were anti−μ to cross link and activate the BCR and H_2_O_2,_ a mild oxidant produced naturally by healthy B cells to control the strength of antigen receptor signaling by reversible inhibition of tyrosine phosphatases [5], [16] [15] The 10-minute time point for modulator treatment was chosen based on kinetic analyses (data not shown) and produced robust, but not necessarily maximal phosphorylation, of all the BCR pathway signaling molecules under study. The H_2_O_2_ concentration chosen was one in which minimal effects were seen on intracellular signaling molecules in healthy B cells (Figure S2) and was consistent with H_2_O_2_ concentrations used in other studies [5], [7], [16], [40].

Consistent with previous reports, anti−µ-mediated BCR signaling was observed and further potentiated by H_2_O_2_ in B cells from healthy donors (Figure S2) [16].

**Figure 2:**
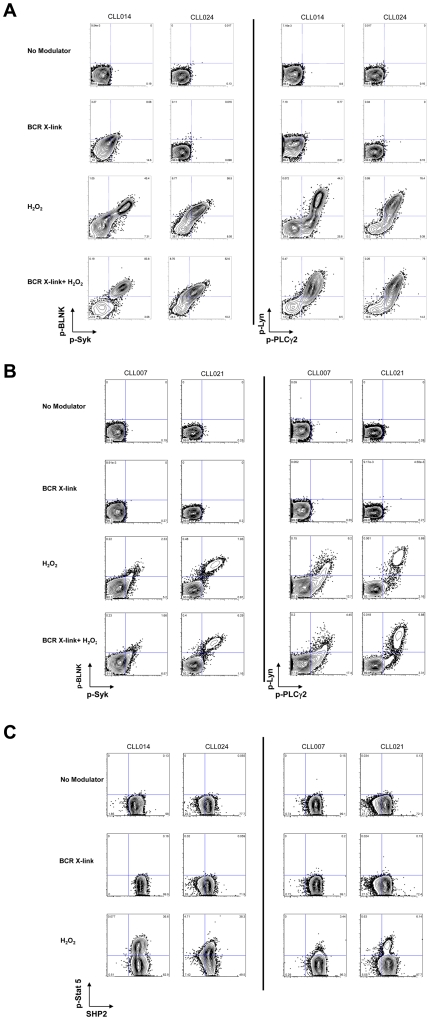
H_2_O_2_ treatment segregates CLL samples into two sub-groups based on magnitude of their signaling responses. (A) CLL B cells were untreated or exposed to anti–µoranti–γalone, H_2_O_2_ alone or the combination for 10 minutes. Representative 2D flow plots show CLL B cell subsets which exhibit (A) robust H_2_O_2_–mediated signaling (B) a reduced H_2_O_2_–mediated signaling response for proximal BCR signaling molecules_._ (C) phosphorylation of Stat5 demonstrates either an increased (left-hand columns) or a marginal response (right-hand columns) to H_2_O_2_ treatment. The 2D plot has SHP-2 along the X-axis as the SHP-2 antibody was in the same antibody panel.

Analysis of the signaling responses showed that the CLL sample cohort was minimally responsive to anti–μ treatment but could be broadly segregated into two patient groups based on their responsiveness to H_2_O_2_. In Group l a significant subpopulation of cells was responsive to H_2_O_2_ such that there was an anti–μ-independent increase in phosphorylation of signaling molecules downstream of the BCR (the mean percentage of a cell subset with activated p-Lyn, p-Syk, p-BLNK or p-PLCγ2 population was 51%, 52% and 45% and 68% respectively, (Table 3 and Figure 2(A)). Signaling was coordinated in that all these components of the proximal B cell receptor network were augmented in concert. In all but three cases, the addition of anti–µ did not mediate a further increase in measured signaling responses, consistent with the notion that aberrant phosphatase activity might be regulating signaling downstream of the BCR in CLL.

**Table 3:** BCR and apoptosis responses in CLL and healthy B cells.

Sample Group	CLL Sample	p-Lyn	p-Syk	p-BLNK	p-Stat 5	p-PLC–γ	p-Akt	p-Erk	Apoptosis
Group I	CLL024	80	65	73	47	77	46	88	**+(55)**
Group 1	CLL003	73	74	64	60	81	74	86	**+(25)**
Group 1	CLL008	57	57	47	36	77	67	84	**+(9)**
Group 1	CLL010	56	54	39	30	85	80	90	**+(22)**
Group 1	CLL009	47	63	54	29	79	56	69	**−(1)**
Group 1	CLL014	43	52	52	36	67	59	57	**+(28)**
Group 1	CLL001	40	41	20	34	51	43	52	**+(13)**
Group 1	CLL002	39	39	36	21	56	40	70	**+(15)**
Group 1	CLL016	27	26	17	23	39	56	41	**+(20)**
Group 1	**Mean**	**51**	**52**	**45**	**35**	**68**	**58**	**71**	
Group 1	**SD**	**16**	**14**	**18**	**11**	**15**	**13**	**17**	
Group 2	CLL019	24	29	19	12	40	66	53	**−(4)**
Group 2	CLL004	20	23	19	13	45	31	76	**+(14)**
Group 2	CLL018	20	21	18	15	33	45	61	**+(17)**
Group 2	CLL013	19	25	19	13	37	26	27	**+(31)**
Group 2	CLL012	17	32	23	7	48	61	34	**−(9)**
Group 2	CLL005	15	18	10	9	35	74	41	**−(3)**
Group 2	CLL020	10	5	5	7	33	40	44	**−(1)**
Group 2	CLL023	10	16	14	10	43	87	83	**−(8)**
Group 2	CLL007	8	7	0	4	19	66	40	**−(0)**
Group 2	CLL021	6	8	9	6	7	8	10	**−(0)**
Group 2	CLL011	5	7	5	3	28	68	19	**−(1)**
Group 2	**Mean**	**14**	**17**	**13**	**9**	**33**	**52**	**44**	
Group 2	**SD**	**6**	**9**	**7**	**4**	**11**	**23**	**22**	
Healthy	CON195	4	6	10	3.4	18	51	21	**+(23)**
Healthy	CON196	3	5	6	0.16	12	48	15	**+(21)**
Healthy	CON219	1	3.5	3	0.6	25	44	26	**+(12)**
Healthy	CON240	1	1	3	0.6	4.5	38	10	**+(34)**
Healthy	CON228	1	4	5	1	4	30.5	29	**+(20)**
Healthy	CON202	0.6	3	5	0.6	5	45	17	**+(30)**
Healthy	CON193	0.5	1.2	2	0.3	4	33	6	**+ (42)**
Healthy	**Mean**	**1.6**	**3.4**	**4.9**	**1.0**	**10.4**	**41.4**	**17.7**	
Healthy	**SD**	**1.3**	**1.7**	**2.5**	**1.0**	**7.7**	**7.1**	**7.7**	
	Ramos	50	53	88	38	77	85	64	**+(21)**

Percentage of B cells from CLL and healthy samples that undergo H_2_O_2_-mediated phosphorylation of intracellular signaling molecules. Values are taken from the 2D cytometry plots (Figures S3(A) and (B), S4(B) and (C) and data not shown). Mean and standard deviations are shown for each CLL and healthy sample. CLL samples were segregated into Group 1 and Group 2 based their H_2_O_2_-mediated p-Stat5 response which was statistically significant (p-value 1.6×10^–4^) between the two groups. There was statistical significance for all other signaling molecules between each group (p-values within the range of 3.8×10^–5^–9×10^–3^). The exception was H_2_O_2_-mediated p-Akt signaling for which the p-value was 0.5. Apoptosis responses to *in vitro* F-Ara-A exposure are shown as+for proficient and – for refractory according to the criteria described in the Data Analysis section on page 11. The percentage of cells that are positive for cleaved caspase 3 and cleaved PARP at 48 hours after background subtraction are in parentheses.

In Group ll a greatly reduced subpopulation of cells had a signaling response after exposure to H_2_O_2_ compared to Group l samples. For example, the mean percentage of cells in a subpopulation with activated p-Lyn, p-Syk, p-BLNK or p-PLCγ2 was 14%, 17%, 13% and 33% respectively (Table 3 and Figure 2(B)).

Interestingly, the H_2_O_2_-mediated p-Akt response was similar between the two groups (a mean cell subpopulation of 58% for Group l and 52% for Group ll, Table 3), suggesting that an alternative phosphatase such as the H_2_O_2_–sensitive PTEN [41] is not differentially regulated in CLL and healthy B cells. The mean number of cells with activation of Erk in Group ll was less than in Group I (44% and 71% respectively). In healthy B cells, all signaling molecules except Akt were minimally responsive to H_2_O_2_ treatment alone (Table 3). Given that H_2_O_2_ is a known inhibitor of phosphatase activity, and that phosphatase activation is a physiological regulator of proximal BCR signaling activities [5], [14]–[17], [21]–[23], these data suggest that deregulation of phosphatase activity could explain some of the differences observed between CLL and healthy B cell signaling responses.

Unexpectedly, in 9/23 CLL samples an increase in p-Stat5 was observed in response to H_2_O_2_ within a subset of cells in individual samples in Group 1 (Table 3, Figure 2(C)(left hand panels) and Figure S2(A)). In 11/23 CLL samples a minimal number of cells exhibited a H_2_O_2_-mediated increase in phosphorylated Stat5 and this was also true for healthy B cells (Table 3, Figure 2(C)(right hand panels, Figure S3(B) and (C)). This observation suggests either that there is a significant re-wiring event downstream of ligand-independent BCR signaling or that an alternative pathway is activated in response to H_2_O_2_, and either could be connected to Stat5 activity.

Interestingly, in many patient samples at least two prominent CLL cell populations with unique and definable signaling responses were observed. For example, a sample in which a dominant cell subset demonstrated augmented signaling in response to H_2_O_2_, other subsets could be identified with marginal responses (Figure 2(A and C)). No such distinctions were observed using basal phosphorylation states, underscoring that activation of BCR signaling molecules highlights the differences in pathway biology between and within samples.

### Lack of responsiveness of the Lyn/Syk/BLNK/PLCγ2 signaling proteins to H_2_O_2_ treatment associated with lack of apoptotic response to F-ara-A in CLL B cells

**Figure 3:**
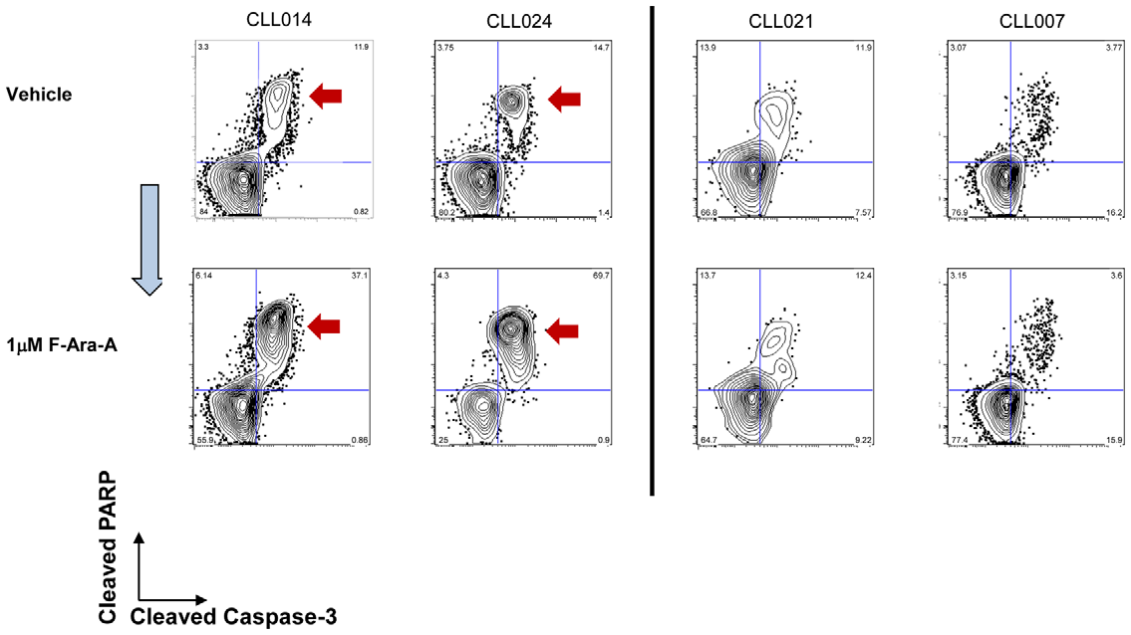
Effect on apoptosis of *In vitro* exposure of CLL B cells to F-ara-A. Representative 2D flow plots (cleaved caspase 3 (X-axis) and cleaved PARP (Y-axis)) show that samples CLL014 and CLL024 undergo F-ara-A-mediated apoptosis (left-hand panels, (red arrows). By contrast samples CLL021 and CLL007 were relatively refractory to F-ara-A treatment (right-hand panels).

There has long been a presumed link between ligand-dependent and independent BCR signaling with B cell survival [17]–[20]. If such links are critical, then it might be further postulated that in CLL and other B cell malignancies, associations may exist between signaling potential downstream of the BCR and apoptotic competence. To test this, apoptotic responses of CLL samples and healthy donors were enumerated by SCNP after *in vitro* exposure to F-ara-A for 48 hours. Representative CLL samples responsive or refractory (Criteria described in Materials and Methods) to *in vitro* F-ara-A exposure show simultaneous measurement of cleaved caspase 3 and cleaved PARP in each cell (Figure 3 and Figure S4(B) (C)). Measurements of loss of mitochondrial cytochrome C in the same cells are consistent with these apoptotic responses (data not shown).

Within apoptotically responsive samples there were at least two cell subpopulations, with a second cell subset that was refractory to *in vitro* F-ara-A exposure (Figure 3 (Left hand panels). This is reminiscent of the signaling data described above in which cell subsets with heterogeneous signal transduction responses were seen within the same sample (Figure 2(A)). At 48 hours 5 leukemic samples (in the absence of F-ara-A) had high background values for cleaved PARP and cytochrome C and were therefore excluded from the analysis (Figure S3). DNA damage was assessed using an antibody against the phospho-threonine 68 epitope on Chk2, the ATM phosphorylation site [42]. Although differences in p-Chk2 levels were seen in cell subsets within F-ara-A responsive and refractory samples, these differences were not statistically significant (data not shown). Furthermore, staurosporine a global kinase inhibitor, and mechanistically distinct from F-ara-A, mediated apoptosis in all except 3 samples (Figure S5(A) and S5(B)).

**Figure 4:**
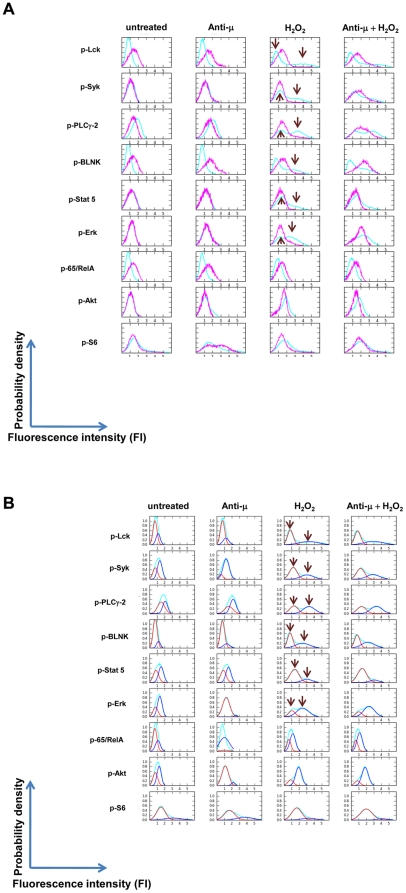
Population distributions of all CLL and all healthy B cells based on their fluorescence intensities. (A) Arcsinh transformed fluorescence intensities either from all gated CLL and healthy B cells in all samples were used to derive the histograms. CLL samples (cyan) demonstrate multiple examples of bimodal activation (arrows), revealed after H_2_O_2_ treatment. By contrast healthy B cells (pink) demonstrate a single cell subset with minimal activation of signaling after H_2_O_2_ treatment. (B) Mixture models comprised of two normal distributions [60] were generated from the histograms of CLL B cells in (A) [61]. These metrics were termed ‘MixMod1’ and ‘MixMod2’ representing the areas under the curve for the distributions with lower (red) and higher (blue) fluorescence intensities, respectively.

Given the pro-survival role BCR signaling molecules play in healthy and tumorigenic B cell biology [5], [12], [16], [43], the data were analyzed for any associations between H_2_O_2_-modulated signaling and apoptotic response to *in vitro* F-ara-A exposure. To evaluate the CLL cohort for trends, all cell events from gated B cells of all CLL samples and, separately, all healthy samples were combined into respective ‘virtual’ samples that represented a composite of the fluorescence intensities for each modulated signaling molecule. The histograms show a greater spread in the fluorescence intensities in CLL versus healthy B cells treated with H_2_O_2_ for each intracellular signaling molecule (Figure 4(A) CLL B cells (cyan) versus healthy B cells (pink)). Combining H_2_O_2_ with anti−µ did not produce additional substantial changes compared to H_2_O_2_ alone in the B cell population distribution of CLL B cells, suggesting that H_2_O_2_ was defining the signaling potential of these CLL B cell populations. This contrasted with healthy B cells in which the combination of H_2_O_2_ and anti−μ, but not H_2_O_2_ alone resulted in an enhanced population distribution based on signaling (Figure 4(A), fourth column [16]).

### Defining cell populations by mixture models in CLL B cells

On the assumption that at least two subpopulations of cells could be driving the distribution of expression in the combined samples, the underlying "subpopulations" were decomposed via mixture modeling for the CLL samples to represent the underlying probability distributions (Figure 4(B)). The trends in the mixture models emphasize the patterns (as expected) of the individual patient samples: the presence of an H_2_O_2_ de-repressed cell subpopulation and a cell subset non-responsive to H_2_O_2_. The mixture model has the benefit of showing, at least for this cohort of patients, the averaged boundaries of where such subpopulations of cells lay on the histograms. The relative numbers of cells in each population defined by these curves were used as metrics that may be linked to the presence or absence of these observed cell subsets to apoptosis response.

**Figure 5:**
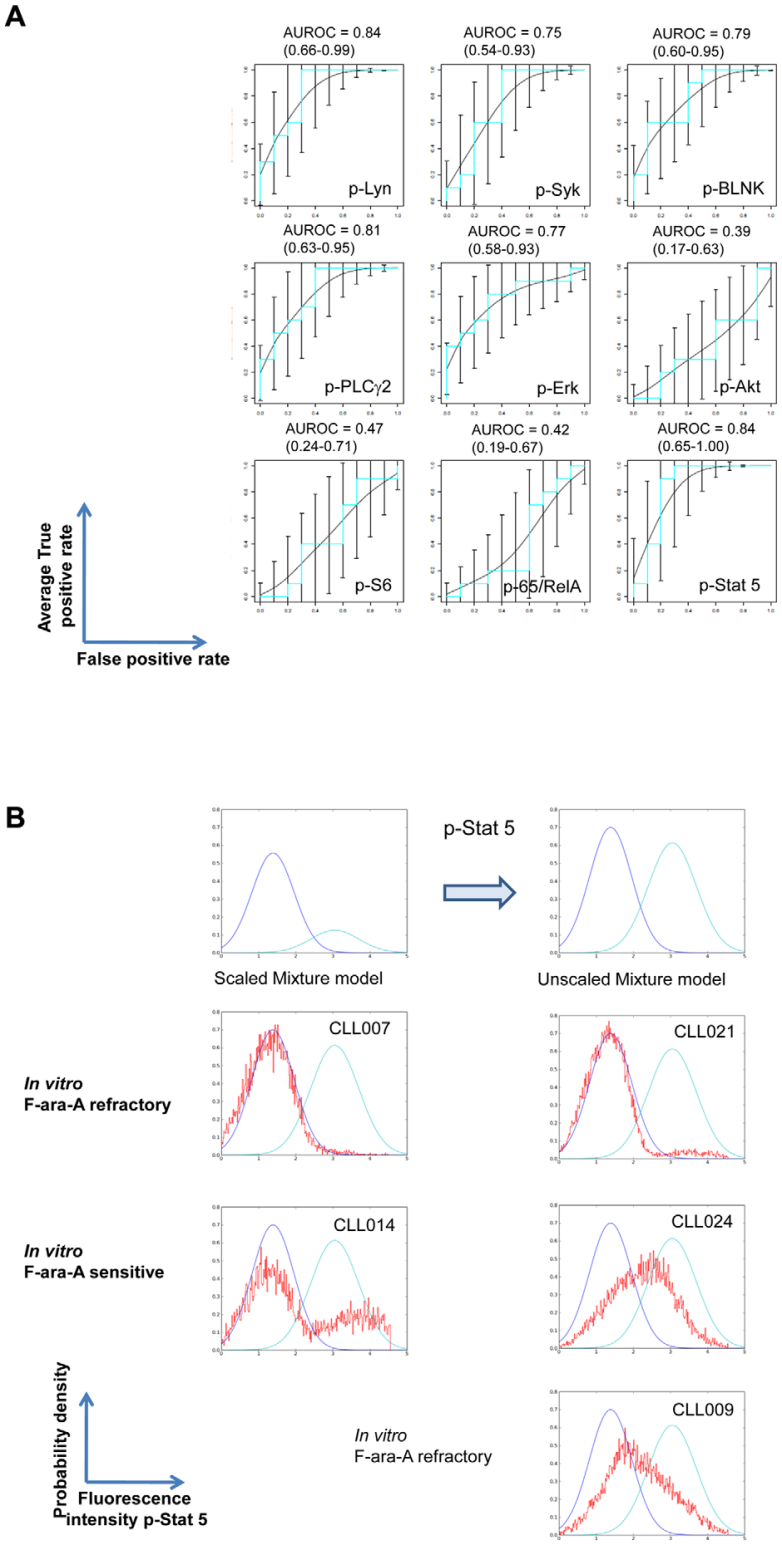
Statistical association between H_2_O_2_-mediated signaling and apoptotic induction by F-ara-A. (A) AUROC curves were expressed with 95% confidence limits in order to evaluate how statistically significant H_2_O_2_–induced signaling is in predicting an *in vitro* apoptotic response to F-ara-A. The mixture model metric for H_2_O_2_-mediated signaling was used to calculate whether there was an association with response or lack of response to *in vitro* exposure to F-ara-A. A value of 0.5 for the ROC plots indicates that the association is due to chance. A value of 1.0 indicates that there is a perfect association. (B) Example of a Mixture Model showing the ability of H_2_O_2_-mediated increase in p-Stat5 to predict response to F-ara-A for an individual patient. An unscaled mixture model was derived from the mixture model for H_2_O_2_-mediated p-Stat5 signaling (top panel and Figure 4B, 5^th^ row). Blue - population density distribution defined by MixMod1, cyan - population density distribution defined by MixMod2, red - population density distribution for H_2_O_2_-mediated p-Stat5 for B cells from an individual patient.

Receiver operating characteristic (ROC) curves (Figure 5(A)) were generated to demonstrate whether presence of either or both of the populations defined by the mixture models (Figure 4(B)) was associated with apoptotic response to *in vitro* F-ara-A exposure. No such associations were observed for healthy B cells, as expected, since the H_2_O_2_ concentration was selected to give no response in healthy B cells as previously reported [16].

Area under the ROC curves (AUC of ROC curve) [44] for signaling induced by H_2_O_2_ treatment showed that p-Lyn (AUC 0.84), p-Syk (AUC 0.75), p-BLNK (AUC 0.79), p-PLCγ2 (AUC 0.81), p-Erk (AUC 0.77) and p-Stat5 (AUC 0.84) signaling stratified patient samples according to their apoptotic pathway response (Figure 5(A)). Using the metrics derived from the mixture models, the ROC curves showed that samples in which H_2_O_2_ exposure revealed signaling were more likely to undergo F-ara-A mediated apoptosis (Figure 4(B), 5(A)). By contrast, samples in which H_2_O_2_ failed to induce signaling were largely non-responsive to F-ara-A (Figures 2(A–C), Figure 3 and Table 3).

Of note, although the range of expression observed for SHP-1, SHP-2 and CD45 tyrosine phosphatases was greater in CLL compared to healthy B cells (Table S2) no association could be seen between their expression with induced signaling or apoptosis. Thus levels of these phosphatases alone were not surrogates for these pathway functions. No associations could be made between the IgV_H_ mutational status or ZAP70 expression status and *in vitro* response to F-ara-A (Fisher’s exact test for association between IgV_H_ status and apoptosis F-ara-A responder/F-ara-A refractory, p value = 1, odds ratio = 0.84).

The areas under the ROC curves demonstrated significant associations between H_2_O_2_-mediated signaling and apoptotic proficiency for the entire CLL sample cohort (Figure 5(A)). However, in order to predict response to *in vitro* F-ara-A treatment for an individual sample, an un-scaled mixture model of for example, H_2_O_2_-induced phosphorylation of Stat5 was established for all the CLL samples (Figure 5(B), top panels). Samples CLL007 and CLL021 have one population distribution of cells and are refractory to F-ara-A exposure. Samples CLL014 and CLL024 show population distributions of cells that span both subpopulations and CLL B cells and these samples are responsive to F-ara-A exposure. CLL009 has a signaling profile predictive of apoptotic sensitivity but was refractory to *in vitro* F-ara-A. This latter sample does not fit the model presumably due to alternative pathways that confer refractoriness to apoptosis (Figure 5(B)). By contrast CLL013 had a reduced H_2_O_2_-mediated signaling response but yet had a strong apoptotic response (Table 3, Figure S3 and data not shown) suggesting that in this sample a different biology may be driving CLL.

## Discussion

Although, several molecular and cytogenetic lesions have emerged as potential prognostic indicators for CLL many disparities and confounding issues limit their clinical utility [33], [45], [46]. For example, although primary resistance to fludarabine has been shown to occur in patients harboring p53 deletions, a recent study reported that treatment-naïve patients with p53 deletions exhibit clinical heterogeneity with some patients experiencing an indolent course [47], [48]. These published clinical studies suggest that there are underlying differences in CLL biology, which if understood, could provide more reliable prognostic information for individual patients.

The data in this study have highlighted a link between H_2_O_2_-induced changes in phosphorylation of signaling proteins downstream of the BCR and *in vitro* F-ara-A-mediated apoptosis in CLL B cells. Specifically, the data showed: (1) The sample cohort could be divided into two groups based on the size of a cell subset within each sample that was responsive to the reactive oxygen species H_2_O_2_ based on increased phosphorylation of p-Lyn, p-Syk, p-BLNK, p-PLCγ2 and p-Stat5. (2) In most cases in which the H_2_O_2_ responsive subpopulation was greater than 30%, a cell subset proficient for F-ara-A mediated apoptosis was seen in the same sample. (3) A mixture modeling metric was derived that was linked to the presence or absence of these observed cell subsets to apoptosis response. (4) AUC values for this model were above 0.75 for p-Lyn, p-Syk, p-BLNK, p-PLCγ2 and p-Stat5 and stratified patient samples according to their apoptotic pathway response.

Although the CLL sample cohort was obtained from CLL patients receiving different treatments, it was striking that their H_2_O_2_ response was able to segregate the samples into two groups. The concentration of H_2_O_2_ used in study was in the millimolar range and was chosen based on its ability to mediate a response in leukemic B cells but not in healthy B cells as previously reported [5]. It is difficult to ascertain exact intracellular concentrations of H_2_O_2_ as its production tends to be localized either in the plasma membrane or in endosomes and inactivating antioxidant enzymes prevent indiscriminate oxidation of intracellular molecules by H_2_O_2_ [14], [49]. H_2_O_2_ is produced by NADPH Oxidase (Nox) enzymes that are activated by cell surface receptors including BCR [14], [15], [50]. To function as an intracellular signaling molecule, H_2_O_2_ must be imported into the cytosol and reported intracellular concentrations range from micromolar to millimolar levels [49], [51]. As an intracellular second messenger, H_2_O_2,_ results in amplification of receptor tyrosine kinase signaling by transiently and reversibly inactivating tyrosine phosphatases (PTPS) through reversible oxidation of the catalytic cysteine to sulfenic acid [14], [49], [52]. A likely role for PTPs in both ligand-dependent and independent BCR signaling was revealed in several studies. In healthy B cells and follicular lymphoma H_2_O_2_ participates in anti-µ mediated signaling [5], [7], [15], [16]. Other reports showed that Syk was activated by pervanadate/H_2_O_2_ in the absence of BCR crosslinking [14], [21]–[23].

That activated BCR signaling molecules, in the absence of ligand, play an important survival role in CLL and other B cell malignancies is substantiated by recent studies. One study showed that in CLL B cells where Lyn protein is over-expressed, its inhibition by small molecule inhibitors *in vitro* in the absence of a BCR ligand, induced apoptosis [53]. Corroborating these findings, *in vitro* treatment of DLBCL and CLL cells with R406 a small molecule inhibitor of Syk (a substrate of Lyn) also induced apoptosis [25], [26], [54]. More recently, a phase I/II clinical trial of fostamatinib disodium, an oral Syk inhibitor showed clinical activity in CLL and non-Hodgkins lymphoma [27]. Another study showed a negative correlation between the expression of ZAP70 with the phosphorylation state of Syk and a positive correlation between p-Syk with p21^cip^, a cell cycle inhibitor [55]. Further insights into the relationship of phosphatase activity with BCR signaling molecules and apoptosis could be determined by experiments including specific tyrosine phosphatase inhibitors specifically targeting SHP-1 and/or CD45. A priori, such inhibitors would be predicted to promote CLL blast cell survival. Consistent with this hypothesis, ectopic expression of protein tyrosine phosphatase, PTPRO, (silenced in CLL by DNA methylation) increased growth inhibition in response to F-ara-A [34]. In DLBCL, PTPROt was identified as a tumor suppressor with a role in tonic BCR signaling [56]. Furthermore, additional studies will be required to determine whether lymphoid tyrosine phosphatase (Lyp) also known as PTPN22, whose expression was reported to be increased in CLL B cells, plays a role in the response of CLL B cells to therapeutic agents (Negro et al., *Blood* (ASH Annual Meeting Abstracts) 2009 114: Abstract 800).

Although not definitively proven, the data in this study potentially support a mechanism whereby H_2_O_2_ through inhibition of tyrosine phosphatases relieves a negative feedback loop that results in activation of signaling proteins within the BCR network. Regardless of its exact mechanism of action, H_2_O_2_ was able to reveal differential signaling within CLL samples and these signaling differences appear to be associated with a signaling posture that either drives, or is driven by the ability of these cells to undergo apoptotic induction by, in this case F-ara-A.

In this study, SCNP analysis, combined with mixture modeling identified at least two phenotypes of CLL B cells based on their H_2_O_2_ – mediated response of signaling molecules (Figure 2(A), (B), (C), Table 3). Notably, some samples demonstrated simultaneous presence of both cell subsets, suggesting co-evolution of signaling phenotypes, a common precursor of these cell subsets, or a lineage relationship between the two subpopulations of cells (Figure 2(A), (C)). Interestingly, and in contrast to studies where the presence of ZAP70 and unmutated IgV_H_ correlated with greater anti−μ-mediated-BCR signaling [25], [29], the signaling responses described here were unrelated to the IgV_H_ mutational status or to ZAP70 expression and spanned a range of cytogenetic abnormalities. Although further studies are warranted to investigate this issue, it is important to note that the above studies [25], [29] were accomplished via indirect assay of total phospho-tyrosine on signaling proteins. In our study, we undertook direct assay of phosphorylation sites using antibodies directed against known, functional, epitopes on a per cell basis. Additionally, no associations were observed between SHP-1, SHP-2, CD22 or CD45 expression levels with H_2_O_2_-mediated signaling (data not shown).

Although not a member of the canonical BCR signaling network, the increase seen in H_2_O_2_ –mediated p-Stat5 could be due to a bystander effect resulting from phosphatase inhibition with consequent increases in kinase activities for which Stat5 is a substrate. Interestingly, Sattler et al, showed the importance of H_2_O_2_ generation with consequent increases in p-Stat5 in several hematopoietic growth factor cascades in cell lines [40]. A pivotal role was also demonstrated for activated Stat5 in hematopoietic stem cell self- renewal and expansion of multi-potential progenitors in myeloid disease [57]. In addition, highly distinctive cytokine responses in Stat5 phosphorylation were reported in both normal and leukemic stem/progenitor cells [58]. Furthermore, in a recent study phospholipase C-β3 was shown to be a tumor suppressor by acting as a scaffold for simultaneous interaction with p-Stat5 and SHP-1 and by doing so promoted the dephosphorylation of p-Stat5 [59]. Whether these mechanisms regulate p-Stat5 in CLL awaits further study.

The clinical complexity (and unpredictability) of CLL as well as the many components governing cell proliferation and survival mechanisms, suggest a diversity of mechanisms that give rise to CLL. Nonetheless, the current studies, although mechanistically incomplete demonstrate a convergence of signaling patterns in CLL that lead to a remarkably limited set of phenotypic cell signaling outcomes. This suggests that despite the underlying molecular and clinical heterogeneity that maintains cellular homeostasis in CLL, only a limited number of signaling pathway variations exist and these may be exploited for therapeutic benefit. Although the sample set was limited, the encouraging AUC values (Figure 5(A)) endorse follow-up studies with expanded sample cohorts, both cryopreserved and fresh, to determine whether SCNP of individual samples can predict treatment outcome and stratify patients who might gain the most benefit from fludarabine-based treatment regimens.

## Supporting Information

Figure S1 **Gating scheme applied to B cells from CLL and healthy donors.**
(TIF)

Figure S2 **H_2_O_2_ amplifies BCR-mediated signaling in healthy B cells.** PBMCs from healthy donors were either untreated or stimulated for 10 minutes with anti-µalone, H_2_O_2_ alone or the combination. 2D flow plots of gated B cells show exemplary samples in which H_2_O_2_ potentiates anti-µ mediated signaling of proximal BCR effectors as previously reported [16].
(TIF)

Figure S3 **H_2_O_2_ treatment segregates CLL samples into two groups based on p-Stat 5 signaling in cell subsets.** Changes in Stat 5 phosphorylation are shown in 2D flow plots and panels (A) and (B) show samples organized by their apoptotic response to *in vitro* F-ara-A exposure. (A) Stat 5 is phosphorylated in response to H_2_O_2_ alone in a CLL B cell subset within this CLL sample sub-group. All samples with these Stat 5 responsive cells undergo F-ara-A-induced apoptosis. (B) Minimal Stat 5 phosphorylation is seen in response to H_2_O_2_ alone within this CLL sample sub-group. All samples except for CLL009 fail to undergo H_2_O_2_–mediated Stat 5 phosphorylation. (C) Stat 5 is not phosphorylated in healthy B cells in response to H_2_O_2_.
(TIF)

Figure S4 **Measurements of apoptosis after *In vitro* exposure of all samples from CLL and healthy donors to F-ara-A.** (A) 2D flow plots show that healthy B cells undergo apoptosis in response to F-ara-A exposure. (B) 2D flow plots in which CLL B cells subsets undergo apoptosis after exposure to F-ara-A. (C) 2D flow plots in which CLL B cells subsets are refractory to F-ara-A exposure.
(TIF)

Figure S5 **Measurements of apoptosis after *in vitro* exposure of CLL samples to staurosporine (5µM) for 6 hours.** (A) 2D flow plots showing response of samples that were recorded as F-ara-A responders (Table 3 and Figure S4 (A). (B) 2D flow plots showing response of samples that were recorded as F-ara-A non-responders (Table 3 and Figure S4 (B)).
(TIF)

Table S1 (DOCX)

Table S2 (DOCX)
