# Supplementary figures and images for: Association of Reactive Oxygen Species-Mediated Signal Transduction with In Vitro Apoptosis Sensitivity in Chronic Lymphocytic Leukemia B Cells
Source: PLoS One. 2011 Oct 10;6(10):e24592. doi: 10.1371/journal.pone.0024592 (PMC3189964; doi:10.1371/journal.pone.0024592)

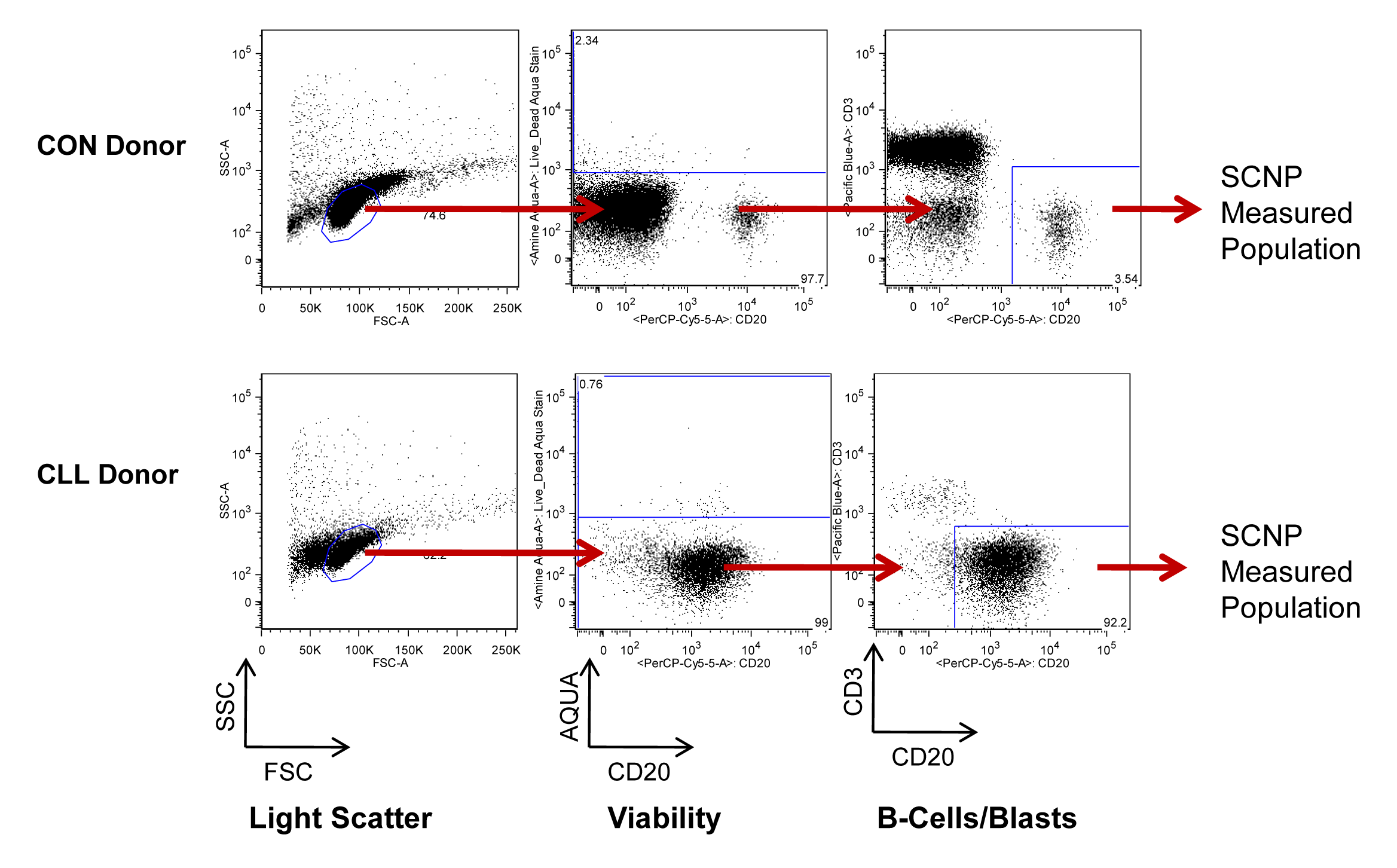

Supplement: Figure S1 — Gating scheme applied to B cells from CLL and healthy donors. (TIF) [file pone.0024592.s001.tif]

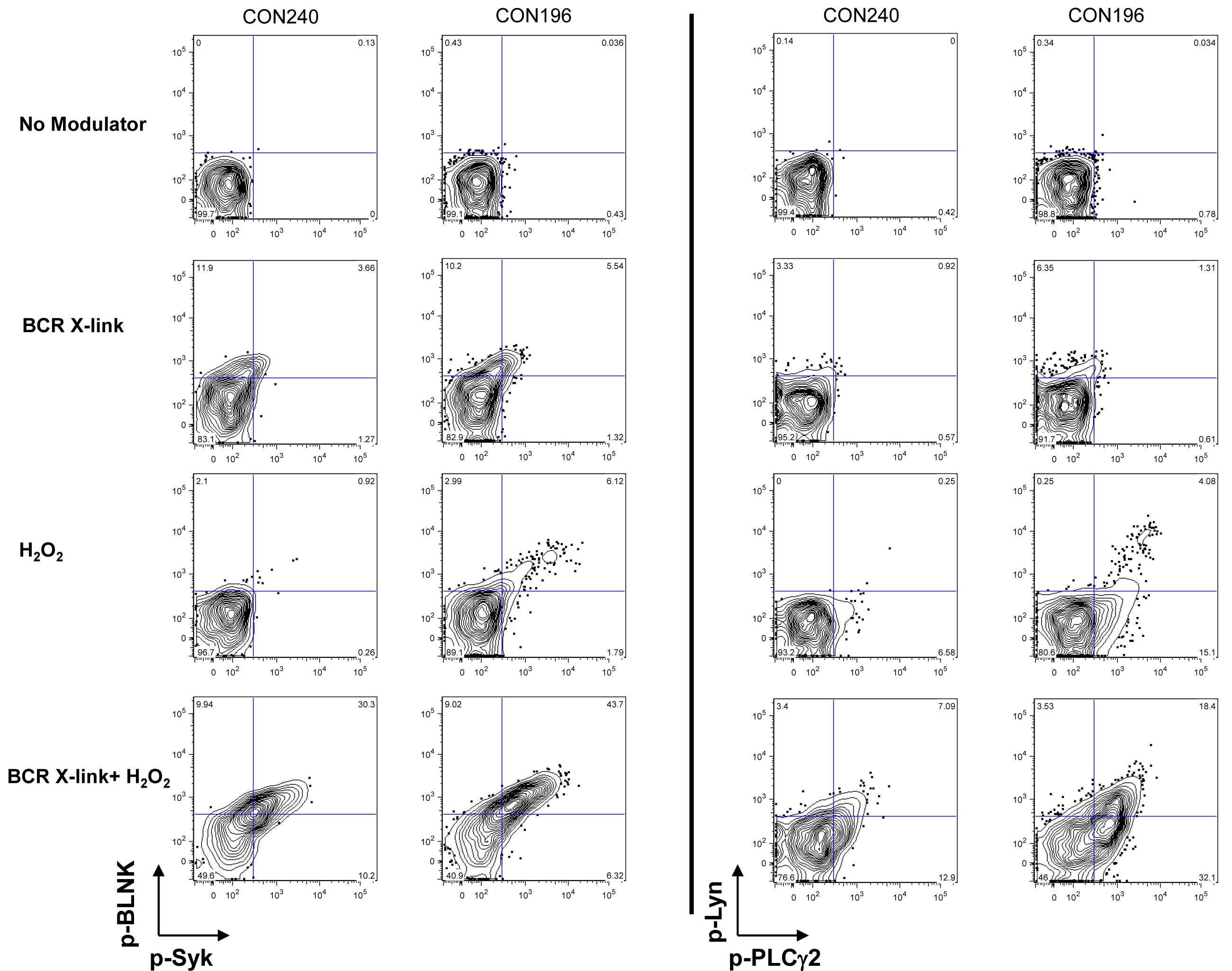

Supplement: Figure S2 — H2O2 amplifies BCR-mediated signaling in healthy B cells. PBMCs from healthy donors were either untreated or stimulated for 10 minutes with anti-µalone, H2O2 alone or the combination. 2D flow plots of gated B cells show exemplary samples in which H2O2 potentiates anti-µ mediated signaling of proximal BCR effectors as previously reported [16]. (TIF) [file pone.0024592.s002.tif]

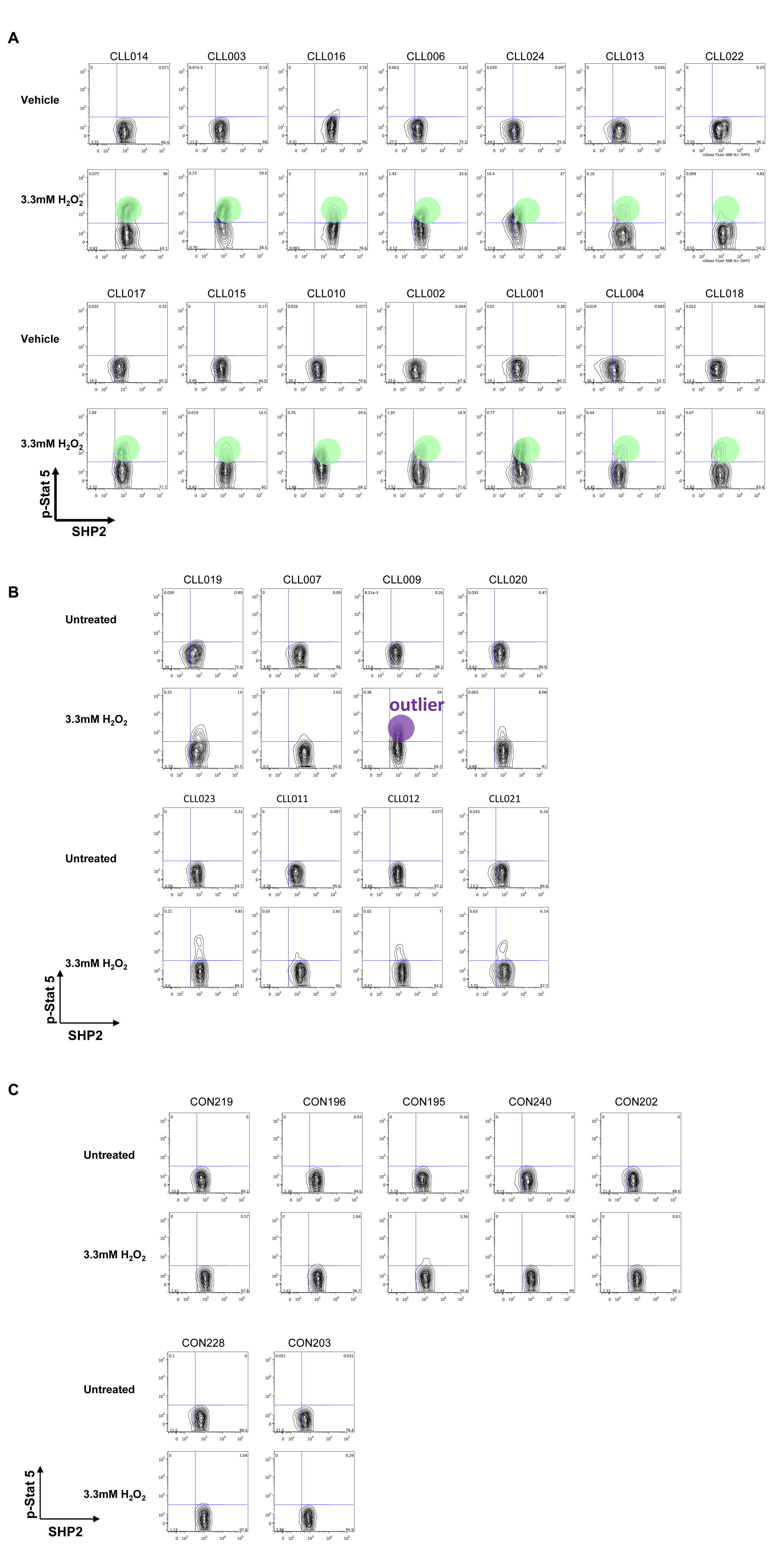

Supplement: Figure S3 — H2O2 treatment segregates CLL samples into two groups based on p-Stat 5 signaling in cell subsets. Changes in Stat 5 phosphorylation are shown in 2D flow plots and panels (A) and (B) show samples organized by their apoptotic response to in vitro F-ara-A exposure. (A) Stat 5 is phosphorylated in response to H2O2 alone in a CLL B cell subset within this CLL sample sub-group. All samples with these Stat 5 responsive cells undergo F-ara-A-induced apoptosis. (B) Minimal Stat 5 phosphorylation is seen in response to H2O2 alone within this CLL sample sub-group. All samples except for CLL009 fail to undergo H2O2–mediated Stat 5 phosphorylation. (C) Stat 5 is not phosphorylated in healthy B cells in response to H2O2. (TIF) [file pone.0024592.s003.tif]

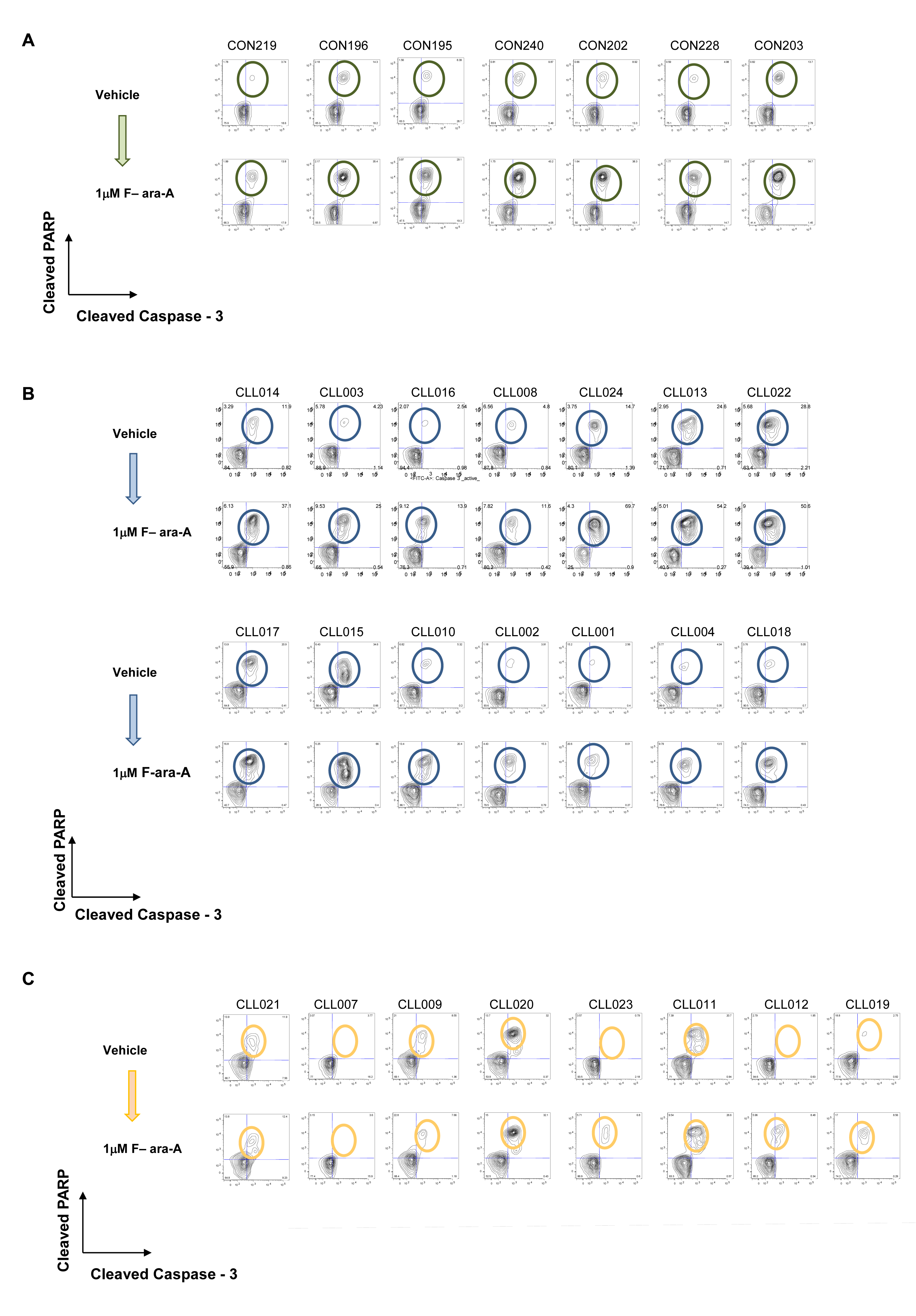

Supplement: Figure S4 — Measurements of apoptosis after In vitro exposure of all samples from CLL and healthy donors to F-ara-A. (A) 2D flow plots show that healthy B cells undergo apoptosis in response to F-ara-A exposure. (B) 2D flow plots in which CLL B cells subsets undergo apoptosis after exposure to F-ara-A. (C) 2D flow plots in which CLL B cells subsets are refractory to F-ara-A exposure. (TIF) [file pone.0024592.s004.tif]

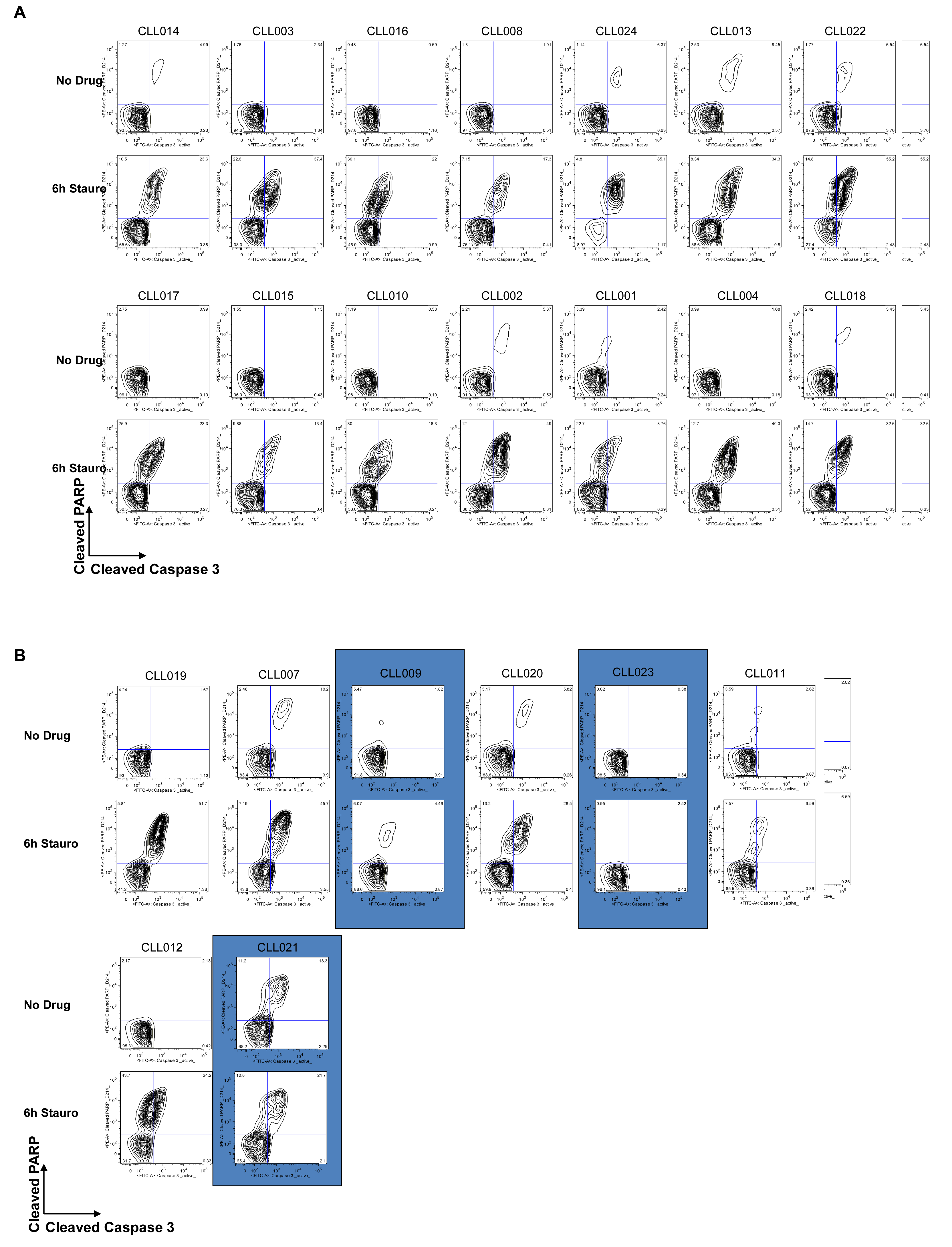

Supplement: Figure S5 — Measurements of apoptosis after in vitro exposure of CLL samples to staurosporine (5µM) for 6 hours. (A) 2D flow plots showing response of samples that were recorded as F-ara-A responders (Table 3 and Figure S4 (A). (B) 2D flow plots showing response of samples that were recorded as F-ara-A non-responders (Table 3 and Figure S4 (B)). (TIF) [file pone.0024592.s005.tif]
