# Supplementary material for: Association of Reactive Oxygen Species-Mediated Signal Transduction with In Vitro Apoptosis Sensitivity in Chronic Lymphocytic Leukemia B Cells
Source: PLoS One. 2011 Oct 10;6(10):e24592. doi: 10.1371/journal.pone.0024592 (PMC3189964; doi:10.1371/journal.pone.0024592)
Supplement: Table S1 — (DOCX) [file pone.0024592.s006.docx]

| \|  \| Ax488 \| PE \| Ax647 \| \| --- \| --- \| --- \| --- \| \| Signaling Panel 1 \| p-Akt(S473)* \| p-Syk(Y352)/p-ZAP70(Y319) \| p-BLNK(Y84) \| \| Signaling Panel 2 \| p-S6(S235/S236)* \| p-PLCγ2(Y759) \| p-LcK(Y505) \| \| Signaling Panel 3 \| p-ErK(T202/Y204) \| Empty \| SHP-1** (2° Goat-anti-rabbit-Ax647) \| \| Signaling Panel 4 \| SHP-2* (2° Goat-anti-rabbitAx488) \| p-Stat 5(Y694) \| p65/RelA(S529) \| \|  \| FITC \| PE \| Ax647 \| \| Apoptosis Panel 1 \| Cleaved Caspase 3 \| Cleaved PARP \| Cytochrome C \| \| Apoptosis Panel 2 \| Empty \| Cleaved PARP \| p-Chk2(T68)* (2° GaR-Ax647) \| \|  \| FITC \| PE \| APC \| \| Phenotypic Panel 1 \| IgM \| IgD \| IgG \| \| Phenotypic Panel 2 \| λ-light chain \| κ-light chain \| CD38 \| \| Phenotypic Panel 3 \| CD45 \| CD79β \| CD22 \| \| B cell gating Panel \| CD3-Pacific Blue \| CD20-PerCPCy5.5 \| CD5-biotin (Q-dot streptavidin) \| |
| --- | --- | --- | --- | --- | --- | --- | --- | --- | --- | --- | --- | --- | --- | --- | --- | --- | --- | --- | --- | --- | --- | --- | --- | --- | --- | --- | --- | --- | --- | --- | --- | --- | --- | --- | --- | --- | --- | --- | --- | --- | --- | --- | --- | --- | --- | --- | --- | --- | --- | --- | --- | --- |
