# Supplementary material for: Association of Reactive Oxygen Species-Mediated Signal Transduction with In Vitro Apoptosis Sensitivity in Chronic Lymphocytic Leukemia B Cells
Source: PLoS One. 2011 Oct 10;6(10):e24592. doi: 10.1371/journal.pone.0024592 (PMC3189964; doi:10.1371/journal.pone.0024592)
Supplement: Table S2 — (DOCX) [file pone.0024592.s007.docx]

|  |  | **IgM** | **IgD** | **IgG** | **CD79b** | **CD19** | **CD20** | **CD5** | **CD38** | **CD22** | **CD45** | **SHP-1** | **SHP-2** |
| --- | --- | --- | --- | --- | --- | --- | --- | --- | --- | --- | --- | --- | --- |
| CLL B cells | mean | 153 | 339 | 14 | 565 | 1390 | 579 | 893 | 241* | 1786 | 14751 | 15502 | 859 |
|  | median | 120 | 226 | 5 | 204 | 800 | 348 | 781 | 17 | 1257 | 13572 | 13221 | 655 |
|  | stdev | 99 | 308 | 86 | 1132 | 1961 | 758 | 610 | 445 | 2162 | 5358 | 9093 | 528 |
| Healthy B cells | mean | 212 | 2788 | -1 | 1428 | 1403 | 1541 | 86 | 2217 | 13165 | 20074 | 19114 | 715 |
|  | median | 219 | 2676 | 2 | 1299 | 1452 | 1391 | 79 | 2346 | 12415 | 19640 | 19045 | 681 |
|  | stdev | 28 | 991 | 7 | 337 | 190 | 354 | 14 | 622 | 2327 | 1446 | 2774 | 75 |
